# Effects of a Pasty Bone Cement Containing Brain-Derived Neurotrophic Factor-Functionalized Mesoporous Bioactive Glass Particles on Metaphyseal Healing in a New Murine Osteoporotic Fracture Model

**DOI:** 10.3390/ijms19113531

**Published:** 2018-11-09

**Authors:** Vivien Kauschke, Maike Schneider, Annika Jauch, Matthias Schumacher, Marian Kampschulte, Marcus Rohnke, Anja Henss, Coralie Bamberg, Katja Trinkaus, Michael Gelinsky, Christian Heiss, Katrin Susanne Lips

**Affiliations:** 1 Experimental Trauma Surgery, Justus-Liebig-University Giessen, Aulweg 128, 35392 Giessen, Germany; maike.schneider33@gmx.de (M.S.); annika@rebske.com (A.J.); Coralie.E.Bamberg@med.uni-giessen.de (C.B.); Katja.Trinkaus@chiru.med.uni-giessen.de (K.T.); Christian.Heiss@chiru.med.uni-giessen.de (C.H.); Katrin.S.Lips@chiru.med.uni-giessen.de (K.S.L.); 2 Centre for Translational Bone, Joint and Soft Tissue Research, Medical Faculty and University Hospital, Technische Universität Dresden, Fetscherstrasse 74, 01307 Dresden, Germany; m.schumacher@maastrichtuniversity.nl (M.S.); michael.gelinsky@tu-dresden.de (M.G.); 3 Currently at MERLN Institute for Technology-Inspired Regenerative Medicine, Maastricht University, 6200 MD Maastricht, The Netherlands; 4 Department of Radiology, University Hospital of Giessen-Marburg, Campus Giessen Klinikstrasse 33, 35392 Giessen, Germany; Marian.Kampschulte@radiol.med.uni-giessen.de; 5 Institute of Physical Chemistry and Center for Materials Research, Justus-Liebig-University Giessen, Heinrich-Buff-Ring 17, 35392 Giessen, Germany; Marcus.Rohnke@phys.chemie.uni-giessen.de (M.R.); anja.henss@phys.chemie.uni-giessen.de (A.H.); 6 Department of Trauma, Hand and Reconstructive Surgery, University Hospital of Giessen-Marburg, Campus: Giessen, Rudolf-Buchheim-Strasse 7, 35392 Giessen, Germany

**Keywords:** osteoporosis, metaphyseal fracture healing model, α-TCP-based HA-forming bone cement, mesoporous bioactive glass, BDNF

## Abstract

The development of new and better implant materials adapted to osteoporotic bone is still urgently required. Therefore, osteoporotic muscarinic acetylcholine receptor M3 (M3 mAChR) knockout (KO) and corresponding wild type (WT) mice underwent osteotomy in the distal femoral metaphysis. Fracture gaps were filled with a pasty α-tricalcium phosphate (α-TCP)-based hydroxyapatite (HA)-forming bone cement containing mesoporous bioactive CaP-SiO_2_ glass particles (cement/MBG composite) with or without Brain-Derived Neurotrophic Factor (BDNF) and healing was analyzed after 35 days. Histologically, bone formation was significantly increased in WT mice that received the BDNF-functionalized cement/MBG composite compared to control WT mice without BDNF. Cement/MBG composite without BDNF increased bone formation in M3 mAChR KO mice compared to equally treated WT mice. Mass spectrometric imaging showed that the BDNF-functionalized cement/MBG composite implanted in M3 mAChR KO mice was infiltrated by newly formed tissue. Leukocyte numbers were significantly lower in M3 mAChR KO mice treated with BDNF-functionalized cement/MBG composite compared to controls without BDNF. C-reactive protein (CRP) concentrations were significantly lower in M3 mAChR KO mice that received the cement/MBG composite without BDNF when compared to WT mice treated the same. Whereas alkaline phosphatase (ALP) concentrations in callus were significantly increased in M3 mAChR KO mice, ALP activity was significantly higher in WT mice. Due to a stronger effect of BDNF in non osteoporotic mice, higher BDNF concentrations might be needed for osteoporotic fracture healing. Nevertheless, the BDNF-functionalized cement/MBG composite promoted fracture healing in non osteoporotic bone.

## 1. Introduction

Physiological bone undergoes constant remodeling which is a balanced process accomplished by bone forming osteoblasts and bone resorbing osteoclasts. In osteoporosis this balance is disturbed in favor of osteoclastic bone resorption. Consequences are low bone mineral density and diminished bone microarchitecture often leading to fractures [1]. Osteoporotic fractures are a severe health problem worldwide with 8.9 million cases every year [1]. Predominant osteoporotic fracture sites are vertebrae, proximal femurs, proximal humeri and distal radii [2]. The second common fracture site in elderly people is the distal femur [3].

Usually osteoporosis is experimentally induced in animal models by ovariectomy which can be accompanied by a deficiency diet lacking vitamins C, D2, D3, calcium and being phosphorus as well as soy- and phytoestrogen-free [4]. Our group showed that mice deficient in M3 mAChR possess low bone mineral density, declined cortical thickness and reduced biomechanical bone strength reflecting characteristics of human postmenopausal osteoporosis [5]. Therefore, we consider this KO mouse strain as an adequate model to analyze osteoporotic fracture healing having the advantage of no prior ovariectomy and deficiency diet.

In osteoporosis, fractures are mostly located in the metaphysis [2]. However, most animal fracture studies have been performed in the diaphysis of femurs or tibias [2]. To better understand fracture healing of osteoporotic bone, osteotomies should be performed in the metaphyseal region, particularly at typical osteoporotic fracture sites. It is known that in osteoporotic fractures recruitment of mesenchymal stem cells (MSCs) and their differentiation are impaired [2]. Thus, osteoporotic bone requires materials which stimulate osteoblast differentiation. Calcium phosphate cements (CPCs) are preferred substitute materials for bone defects because of their osteoconductive, resorbable and biocompatible characteristics [6]. Moreover, in osteoporosis additionally stimulating factors are necessary for the differentiation of MSCs into osteoblasts [7]. CPCs can serve as carriers for substances that stimulate osteogenic differentiation of MSCs. Schumacher et al. (2013) showed that strontium incorporated in CPC increased MSCs proliferation and their osteoblastic differentiation [8]. Growth factors like bone morphogenic protein (BMP), vascular endothelial growth factor (VEGF) or transforming growth factor beta (TGF-ß) are beneficial for bone regeneration [9]. Hence, there have been attempts to load growth factors into CPCs to allow local release into the defect area upon implantation [10]. Therefore, MBG particles, among other carriers, have been integrated into CPCs to tailor the growth factor release profile [11]. Another growth factor involved in fracture healing is BDNF [12]. BDNF is a neurotrophin known to be essential for the development of the nervous system. Furthermore, it was shown that BDNF is involved in fracture healing since it was detected in fracture gap tissue but not during physiological bone remodeling [12]. Moreover, BDNF significantly increased new bone formation after applying BDNF to mandibular osteotomies of rats [13]. Therefore, BDNF appears as potential drug suitable to be incorporated in CPCs and to stimulate bone formation during fracture healing in osteoporosis.

## 2. Results

### 2.1. Real-Time Reverse Transcriptase Polymerase Chain Reaction (Real-Time RT-PCR)

**Figure 1:**
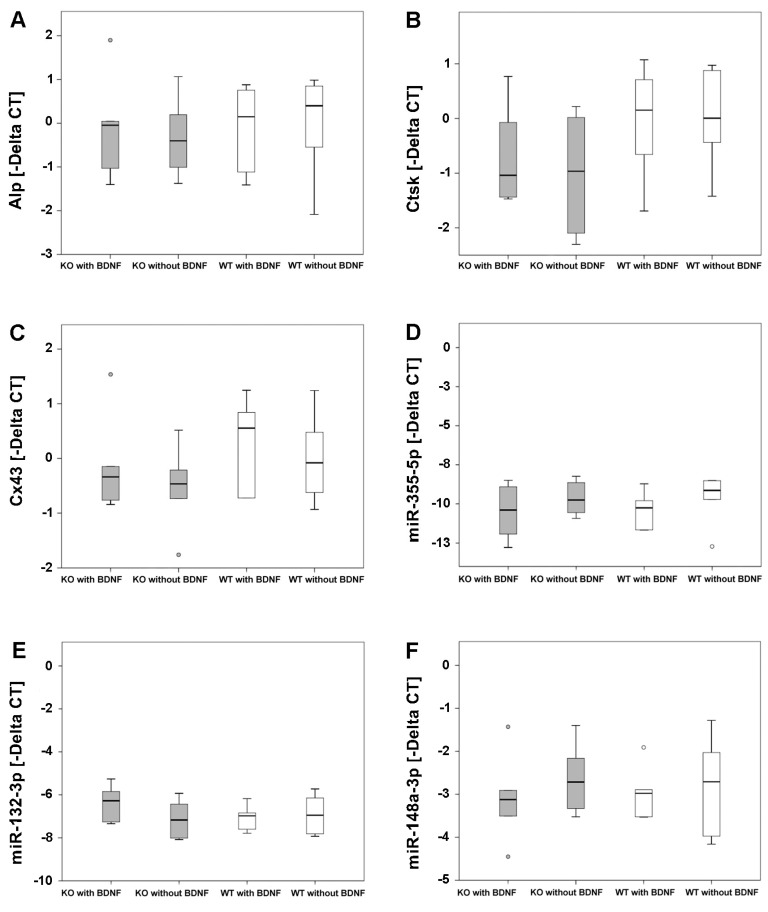

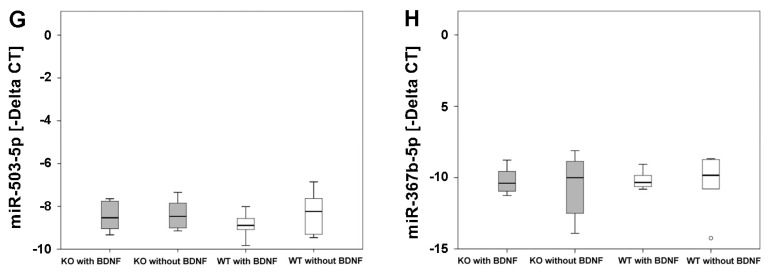
mRNA expression of Alp (**A**), Ctsk (**B**) and Cx43 (**C**), microRNAs miR-335-5p (**D**), miR-132-3p (**E**), miR-148a-3p (**F**), miR-503-5p (**G**) and miR-376b-5p (**H**) at the bone implant interface in femurs of M3 mAChR KO and corresponding WT mice. The grey and white circles indicate outliers.

Alkaline phosphatase (Alp), cathepsin K (Ctsk) and connexin 43 (Cx43) mRNAs were expressed at the bone tissue implant interface in femurs of M3 mAChR KO and WT mice. Whereas an increase in Alp mRNA expression indicates higher osteoblastic activity, up-regulation of Ctsk mRNA expression suggests more osteoclastic activity. Cx43 supports communication between bone cells. However, we determined no statistically significant differences in mRNA expression of Alp, Ctsk and Cx43 between M3 mAChR KO and WT mice that received the pasty cement/MBG composite with or without BDNF (Figure 1A–C) after 35 days of fracture healing.

The microRNAs miR-335-5p, miR-132-3p, miR-148a-3p and miR-503-5p are involved in the regulation of bone remodeling, which were detected at the bone implant interface of femurs of M3 mAChR KO and corresponding WT mice. The same was observed for miR-376b-5p. Delta Cycle Threshold (CT) values of all microRNAs analyzed were not significantly different between all experimental groups (Figure 1D–H).

### 2.2. Micro-Computed Tomography (Micro-CT) Analysis

**Figure 2:**
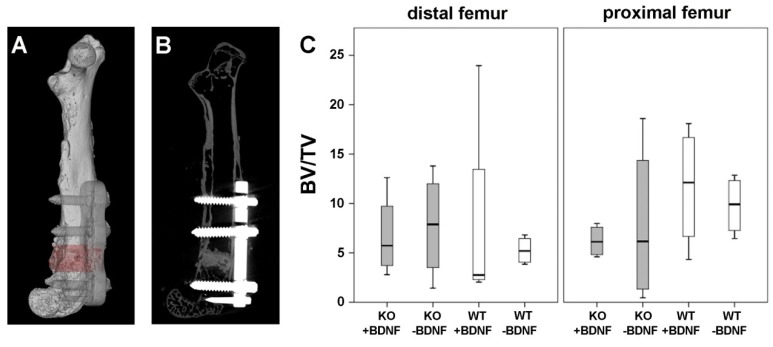
Micro-CT: (**A**) 3D volume rendering of a M3 mAChR KO mouse femur with attached metaphyseal locking plate. Marked in red is the bone cement/MBG composite, (**B**) cross sectional image of the same femur with attached metaphyseal locking plate shown in white (**C**) analysis of BV/TV in M3 mAChR KO and WT mice.

A post-mortem Micro-CT image of the operated femur illustrates the location of the attached metaphyseal MouseFix locking plate and of the bone cement/MBG composite in the metaphysis of the right femur (Figure 2A,B). The success of the surgical procedure is shown in Figure 2A,B. Figure 2A displays a 3D volume rendering of an operated femur of a M3 mAChR KO mouse and Figure 2B a cross sectional image of the same femur.

**Figure 3:**
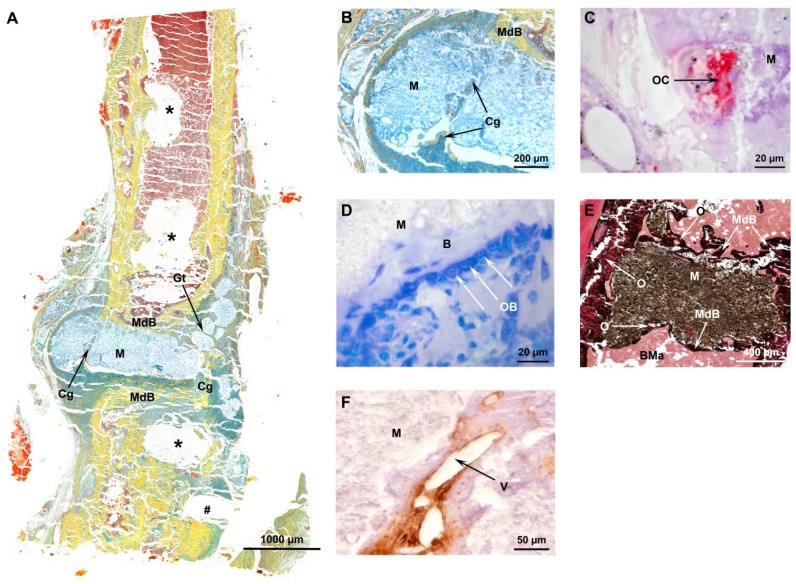
Histology: (**A**) Movat pentachrome staining of a femur section, (**B**) close-up of the same section showing the cement/MBG composite (M), granulation tissue (Gt) in green, cartilage (Cg) in greenish blue and mineralized bone (MdB) in yellow, * point out positions of screws and the **#** depicts the location of the locking pin, (**C**) osteoclast (OC) visualized by TRAP in red, (**D**) osteoblasts (OB) visualized by toluidine blue, B indicates bone tissue (**E**) mineralization visualized by von Kossa/van Gieson in black, (**F**) ASMA detection of a blood vessel (V); O = osteoid, BMa = bone marrow.

Detailed analysis of femurs showed no significant differences in bone volume fraction (BV/TV) of the distal and proximal femur between the different experimental groups, independent of whether the bone cement/MBG composite was functionalized with or without BDNF (Figure 3C).

### 2.3. Histology and Histomorphometry

Each histological technique applied (Movat pentachrome staining, tartrate-resistant acidic phosphatase (TRAP), toluidine blue, von Kossa/van Gieson and alpha smooth muscle actin (ASMA)) revealed that the implant was tolerated and bone formation occurred. Movat pentachrome staining revealed that bone healing progressed as exemplarily shown in Figure 3A. It shows an overview of a femur section of a WT mouse that received the cement/MBG composite functionalized with BDNF. Figure 3B shows details of the same section displayed in Figure 3A. We detected granulation tissue (Gt) but also cartilage (Cg) inside parts of the material as well as newly formed mineralized bone (MdB) surrounding the material, indicating new bone formation and osteointegration (Figure 3A,B).

TRAP detection showed osteoclasts resorbing the material (Figure 3C) and osteoblasts forming new bone at the implant interface (Figure 3D) implanted in a M3 mAChR KO mouse.

Von Kossa/van Gieson staining confirmed the results of Movat pentachrome staining that mineralized tissue was formed at the implant interface, visualized in black (Figure 3E).

Moreover, ASMA immunohistochemistry depicted blood vessels within the material (Figure 3F), indicating vascularization of newly formed bone.

**Figure 4:**
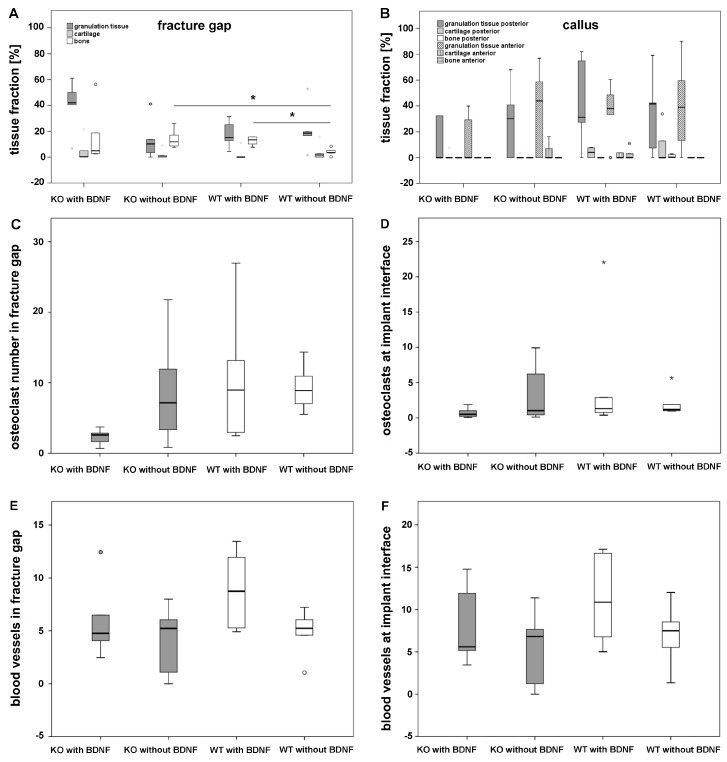
Histomorphometrical analysis: fraction of granulation tissue, cartilage and bone in fracture gap (**A**) and in callus, the dotted line represents boxplots indicating a value of zero (**B**), osteoclast numbers within the fracture gap (**C**) and at implant interface (**D**), number of blood vessels in fracture gap (**E**) and at implant interface (**F**). The grey and white circles indicate outliers. The grey * indicate extreme outliers. The black * illustrate statistically significant differences with a likelihood of *p* ≤ 0.05.

Histomorphometrical analysis showed a significant increase of newly formed bone at the implant interface of WT mice that received the BDNF-functionalized cement/MBG compared to WT mice that received the cement/MBG composite without BDNF. Moreover, a significant increase of newly formed bone was observed in M3 mAChR KO mice that were treated with the cement/MBG composite without BDNF compared to WT mice that received the same material. Granulation tissue and cartilage were not significantly altered between all experimental groups (Figure 4A). In callus no significant differences in tissue fraction of granulation tissue, cartilage and newly formed bone were measured in all experimental groups (Figure 4B).

Osteoclast count based on TRAP detection revealed no significant differences between differently treated KO and WT mice within the fracture gap (Figure 4C) or at the implant interface (Figure 4D). Moreover, there were no significant differences in the number of blood vessels within the fracture gap (Figure 4E) or at the implant interface (Figure 4F) in all experimental groups.

### 2.4. Time-Of-Flight Secondary Ion Mass Spectrometry (ToF-SIMS), High-Resolution Scanning Electron Microscopy (HR-SEM) and Energy-Dispersive X-ray Spectroscopy (EDS)

**Figure 5:**
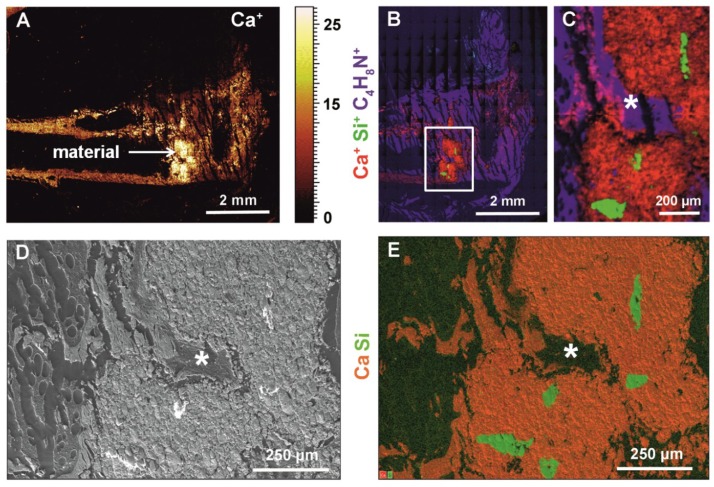
ToF-SIMS, HR-SEM and EDS images of bone cross sections: (**A**) ToF-SIMS overview image of calcium mass distribution within the femur and cement/MBG composite, the lighter the color the more calcium is present indicated by the scale next to the image (**B**,**C**) ToF-SIMS mass images showing calcium (red, Ca^+^), collagen (blue, C_4_H_8_N^+^) and silicon (green, Si^+^), (**C**) close-up of cement/MBG composite indicated with a white box in B, the different colors of the element symbols at the margin of figures B, C and E correspond to colors seen in images, (**D**) HR-SEM image, (**E**) EDS mapping of the cement/MBG composite in red and MBG particles in green. * in C, D and E indicate newly formed bone within a pore that developed after MBG particles dissolved.

Using ToF-SIMS we showed newly formed tissue that grew inside pores within the cement/MBG composite that were formed by MBG after it dissolved (Figure 5). In Figure 5A an overview mass image of calcium distribution in the femur and cement/MBG composite is shown. The lighter the signal the more calcium is present. MBG particles that were not encapsulated by the cement and in contact with the bone marrow were dissolved completely. Silicon from the dissolved MBG was found in the vicinity of the remaining cement. Calcium signal complies with the cement/MBG composite and newly formed bone. Moreover, type I collagen (represented by the mass fragment C_4_H_8_N^+^, confer [14]) with slight amounts of calcium was detected which can be interpreted as newly formed bone tissue as well, since collagen is the main organic component of bone (Figure 5B,C). Figure 5D,E show the corresponding HR-SEM and EDS images, respectively. EDS mapping confirms the mass spectrometric data shown in Figure 5B. Due to the fact that EDS information is obtained from subsurface regions (10 µm and more), the particle shape is slightly different. However, most of the cement was not degraded and not replaced by new bone after 35 days.

### 2.5. Fluorescence-Activated Cell Sorting (FACS) Analysis

**Figure 6:**
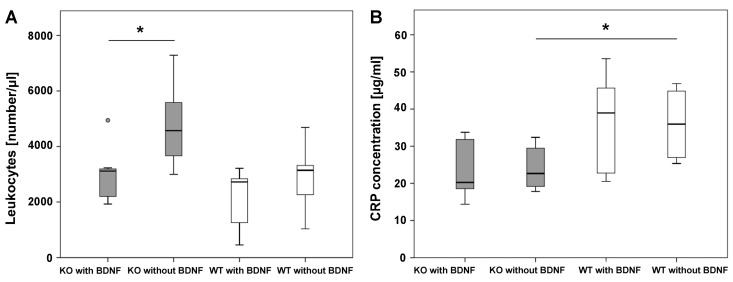
Leukocyte numbers in blood (**A**) and CRP concentration in plasma (**B**). The small grey circle indicates an outlier. The * illustrate statistically significant differences with a likelihood of *p* ≤ 0.05.

FACS analysis of blood showed that leukocyte numbers were significantly lower in M3 mAChR KO mice that received the pasty cement/MBG with BDNF when compared to M3 mAChR KO mice that were implanted with the same material but without BDNF. Between the differently treated WT mice no significant differences were detected in leukocyte numbers. Leukocyte numbers between M3 mAChR KO and WT mice differed not significantly, independent of the material that they were implanted with (Figure 6A).

### 2.6. CRP Analysis

During inflammation CRP is increased. In M3 mAChR KO mice that received the pasty cement/MBG composite without BDNF, CRP concentration was significantly lower at the end of the experiment when compared to WT mice that received the same material. No significant differences were detected when comparing M3 mAChR KO mice which obtained the pasty cement/MBG composite functionalized with BDNF to WT mice that were treated the same. Comparing the M3 mAChR KO mice that were treated with the cement/MBG composite either functionalized with or without BDNF, no significant differences were detected in CRP concentration. The same was observed between differently treated WT mice (Figure 6B).

### 2.7. Analysis of Total Protein as well as Alkaline Phosphates (ALP) Concentration and Activity in Callus Tissue Cultured In Vitro

**Figure 7:**
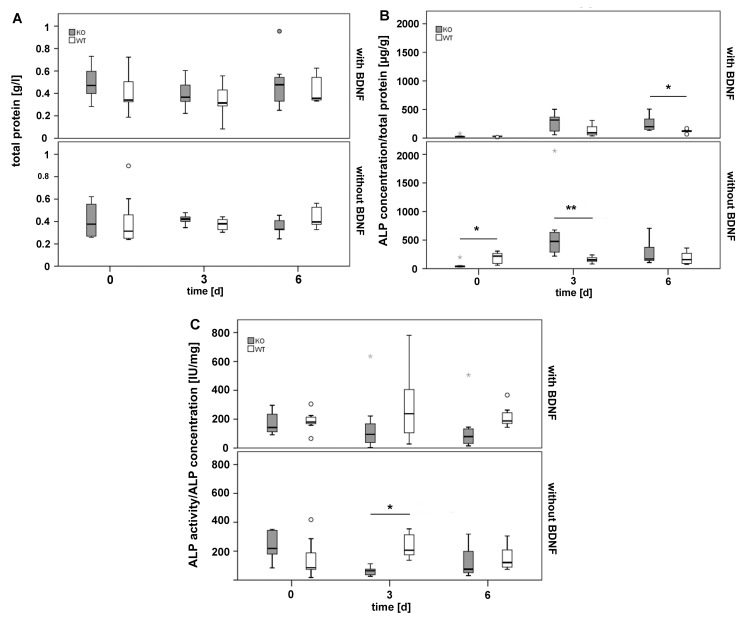
Total protein (**A**) ALP concentration (**B**) and ALP activity (**C**) in callus tissue of M3 mAChR KO and WT mice. The grey and white circles indicate outliers. Extreme outliers are represented by a grey star. The * and ** illustrate statistically significant differences with a likelihood of *p* ≤ 0.05 and 0.01, respectively.

The amount of total protein was similar in callus tissue of all experimental groups (Figure 7A) and served as reference for the calculation of the ALP concentration.

At the beginning of the experimental procedure ALP concentration was significantly higher in callus tissue of WT mice that received the cement/MBG composite without BDNF when compared to M3 mAChR KO mice that were treated the same. In contrast, after 3 days (d) the same material caused a significant increase in ALP concentration in callus tissue of M3 mAChR KO mice. In presence of the BDNF-functionalized cement/MBG composite the ALP concentration was not different between M3 mAChR KO and WT mice after 0 and 3 days. However, after 6 days ALP concentration was significantly higher in M3 mAChR KO mice compared to WT mice (Figure 7B).

Although ALP concentration was significantly higher after 3 days in M3 mAChR KO mice that were implanted with the cement/MBG composite without BDNF, their ALP activity was significantly lower compared to WT mice that were treated the same (Figure 7C).

## 3. Discussion

Our experimental approach delivered for the first time a fracture healing model for testing pasty bone substitute materials in metaphyseal fracture gaps of mice femurs. In this study, a composite based on a pasty α-TCP HA-forming cement and MBG particles functionalized either with or without BDNF was used as bone substitute material, but other pasty bone substitute materials are applicable in this animal fracture model as well.

When conducting an osteoporosis fracture healing model in animals, it should be considered that in humans osteoporotic fractures mostly occur in the metaphysis. Therefore, osteotomies in animals should coincide. This is an important aspect since differences between diaphyseal and metaphyseal fracture healing have been shown [2]. Analyses of osteoporotic, metaphyseal fracture healing have been performed so far in large animal models as sheep or in small animals like rats [15,16], but not in mice. Up to now gaps and drill hole defects have been inflicted into the diaphysis of femurs of ovariectomized or sham operated mice [17]. However, drill hole defects or diaphyseal fractures do not depict the clinical situation of osteoporotic fractures.

Often, osteoporotic fractures occur in the femoral neck [18]. Our animal model does not allow a surgical approach of the proximal femur because of unpractical accessibility of this region and possible damage of tendons which would essentially restrict mice’s motion. Even though, metaphyseal osteotomy using the piezosurgery technique creates defects similar to fractures seen in the clinic, it can cause high temperatures in the defect area which might lead to cell death at the cut edge [19]. Still, piezosurgery is the preferential technique since osteocyte viability was significantly higher when compared to bur defects [20].

In rats osteoporosis has either been induced by ovariectomy alone [21] or accompanied by a diet deficient in vitamins C, D2, D3, calcium, phosphorus and soy- and phytoestrogen-free [4]. In large animal models like sheep osteoporosis can be similarly induced [22]. However, there are osteoporosis animal models that do not require ovariectomy or deficiency diet, like the scenescence-accelarated mouse (SAM) strain 6 (SAMP6) which is used as model for senile osteoporosis [23]. In our model mice were osteoporotic due to the KO of M3 mAChR. Additional surgery or feeding a deficiency diet is not necessary for osteoporosis induction.

Osteoporotic fractures of the femur heal slower than non osteoporotic fractures [24]. Therefore, fracture healing in osteoporosis often requires bone substitute materials functionalized with drugs to stimulate bone formation [25]. CPCs are commonly used materials in the clinic. They are preferred because of their osteoconductive characteristics and relatively fast setting reactions taking place at low temperature. The low temperature allows loading with growth factors making CPCs also eligible as drug delivery systems [9]. Another advantage of CPCs is that they are resorbable [26]. The paste-like α-TCP that served as matrix material in the composite used in our study can be actively resorbed by osteoclast-like cells and subsequently be replaced by new bone [27]. By addition of MBG particles to the cement, a composite was obtained that allows integration of growth factors into the cement [28]. In our study, MBG particles were loaded with BDNF expecting that integration into the composite might increase bone formation since this growth factor was found in human fracture healing tissue but not during physiological bone remodeling [12]. Moreover, MBG particles support the degradation process by leaving pores within the material after particles have dissolved [11]. We showed that osteoclasts started to resorb the material. However, most of the cement/MBG composite was still present at the end of the experiment which could be changed by adding more MBG particles to the cement. Extrapolating this system to humans, the material could be implanted at different fracture sites, not only femurs. Since the α-TCP HA-forming cement has a pasty consistency at first, the material can be adapted to other bone fractures. BDNF within the cement may directly affect osteoblast activity within the defect area and eventually increase new bone formation, preferentially in osteoporosis. Besides other cell types, BDNF is also expressed by platelets [29,30]. Thus, BDNF could be admixed to the cement using autologous blood to support fracture healing.

Up-regulation of Alp, Ctsk and Cx43 mRNAs indicates expression of important proteins of bone metabolism reflecting bone formation by osteoblasts, bone resorption by osteoclasts and cell communication, respectively. The microRNAs miR-335-5p and miR-132-3p as well as miR-148a-3p and miR-503-5p are involved in the regulation of osteoblast differentiation and osteoclast activity, respectively [31,32,33]. Zhang et al. (2011) showed that osteogenic differentiation was supported by miR-335-5p as a result of down-regulation of Dickkopf-related protein 1 (DKK1) [31]. In contrast, miR-132-3p inhibited osteoblast differentiation [32]. MicroRNA miR-148a-3p was significantly up-regulated during osteoclastogenesis, promoting differentiation of osteoclasts [33]. Overexpression of miR-503-5p prevented osteoclastogensis by inhibition of Receptor Activator of Nuclear Factor Kappa B (NF-κB) Ligand (RANKL) [33]. Our real-time reverse transcriptase polymerase chain reaction (real-time RT-PCR) results showed that mRNA of Alp, Ctsk and Cx43 as well as the microRNAs miR-335-5p, miR-132-3p, miR-148a-3p, miR-503-5p and miR-376b-5p were expressed at the bone implant interface in femurs of mice. However, no significant differences were detected between the different experimental groups. Pan et al. (2012) ascertained that miR-376b-5p inhibits BDNF expression by binding to its gene. In heart tissue inhibition of BDNF expression by miR-376b-5p supported myocardial ischemia injury [34]. Moreover, the M3 mAChR acts cardio-protective [35] and supports bone formation [36]. Since BDNF was also detected in fracture gap tissue and not in physiological bone tissue, presumably BDNF has protective effects in damaged bone as well. Thus, up-regulation of miR-376b-5p at the bone implant interface of M3 mAChR KO and WT mice could promote down-regulation of BDNF and interfere with fracture healing. However, our results showed that miR-376b-5p was neither significantly up- nor down-regulated in M3 mAChR KO and WT mice implanted with the composite with or without BDNF.

Histomorphometrical analysis showed that significantly more new bone was formed in M3 mAChR KO mice that received the pasty cement/MBG composite without BDNF when compared to WT mice that received this material as well. In opposite, WT mice that were implanted the BDNF-functionalized cement/MBG composite showed a significant increase of newly formed bone when compared to WT mice that received the pasty cement/MBG composite without BDNF. Even though significantly more bone formation was observed in the M3 mAChR KO mice group that received the cement/MBG composite without BDNF compared to WT mice that received the same material, ToF-SIMS revealed that formation of new bone took place at the interface and inside the BDNF-functionalized cement/MBG composite after implantation into M3 mAChR KO mice. Generally, ToF-SIMS showed that addition of MBG particles led to faster cement degradation. Nevertheless, the amount of MBG should be increased since most of the material was still present at the end of the experiment. Still, the cement/MBG composite was resorbed by osteoclasts and new bone formed by osteoblasts.

Different effects of BDNF in M3 mAChR KO and WT mice might either be explained by BDNF having bimodal effects or that in M3 mAChR KO mice higher concentrations of BDNF are needed to enhance fracture healing. In vitro a concentration of 40 ng/mL increased osteogenic differentiation of human MSCs of osteoporotic donors [37]. In the introduced mouse model only 21 ng of BDNF were released into each animal, most probably due to less amounts of the carrier MBG. Moreover, BDNF release from the material seemed not to be sufficient.

However, FACS analysis revealed that leukocyte numbers were significantly lower, but within a physiological range, in M3 mAChR KO mice that received the cement/MBG composite with BDNF indicating anti-inflammatory effects of BDNF. This was also observed by Xu et al. (2017) who showed that BDNF suppressed a variety of pro-inflammatory factors [38].

CRP is an acute phase protein and concentrations are high during inflammation. In orthopedic surgery it is considered the preferential biomarker to detect inflammatory processes [39]. In the first days after trauma CRP levels increase but decrease under physiological conditions within the healing process. If the healing process is disturbed, for example due to bacteria, CRP levels rise [40]. Moreover, biocompatibility of the material can be assessed using this biomarker. Still, CRP concentrations also increase when there is an infection at other areas in the body. However, if CRP is not increased, we can conclude that the surgery did not cause an inflammation in mice. Our results showed that M3 mAChR KO mice that received the pasty cement/MBG composite without BDNF revealed significantly lower CRP concentrations in plasma than corresponding WT mice that were treated the same. However, in presence of BDNF no significant differences were measured in CRP levels between M3 mAChR KO and WT mice.

In callus tissue ALP concentrations were significantly higher in M3 mAChR KO than in WT mice after 3 and 6 days in presence of the cement/MBG composite either with or without BDNF. This is an interesting result since in osteoporotic bone tissue, unlike serum, ALP usually is not increased [41] which indicates a stimulating effect of the cement/MBG composite. However, ALP activity was significantly lower in M3 mAChR KO mice than in WT mice that received the cement/MBG composite without BDNF after 3 days. This suggests that stimulation of ALP activity by the cement/MBG composite in osteoporotic mice should be increased.

## 4. Materials and Methods

### 4.1. Animals

Female homozygous M3 mAChR KO breeder mice (B6.129S6-CF1-Chrm3^tm1Jwe^) were obtained from Prof. Dr. Juergen Wess (National Institutes of Health (NIH), Bethesda, MD, USA) and bred in the animal facility of the Justus-Liebig-University Giessen, Germany. Corresponding WT mice (C57BL/6N_Tac_) were purchased from Taconic Biosciences A/S (Ejby, Denmark) and allowed to acclimate for 2 weeks prior to surgery. M3 mAChR KO and WT mice were kept under a 12-h light-dark circle with free access to chow and water. Surgeries were performed on anesthetized 16-week-old female homozygous M3 mAChR KO mice and their corresponding WT mice. Mice were osteoporotic due to the KO of M3 mAChR. Additional surgery or feeding a deficiency diet was not necessary for osteoporosis induction. This refines the animal experiment and thus complies with one aspect of the 3R principle of animal testing (Replacement, Reduction and Refinement). Animal experiments were approved 30 March 2015 by the animal welfare officer as well as the regional authority (animal experiment V 54 -19 c 20 15 h 01 GI 20/15 No. 75/2014) and performed according to the NIH regulations for laboratory animal care and the current version of the German law on animal protection.

### 4.2. Bone Substitute Material for Fracture Gap Closure

A composite comprised of a pasty α-TCP-based HA-forming bone cement (InnoTERE GmbH, Radebeul, Germany) [42] and MBG particles were used to fill fracture gaps [28]. MBG particles with a diameter of approximately 200 µm were synthesized based on protocol by Zhu et al. (2008) [43] as described previously [11]. Prior to animal experiments the bone cement paste and MBG particles were γ-sterilized separately. MBG was additionally loaded with 84.1 ng/g BDNF. Control material of the same composition was free of BDNF. Shortly before insertion into fracture gaps a bone cement/MBG composite was formed by thoroughly dispersing 2 wt % MBG particles with the cement paste. The composite set into a solid phase after implantation in the fracture gap within a few minutes after contact with body fluids.

### 4.3. Surgical Procedure

**Figure 8:**
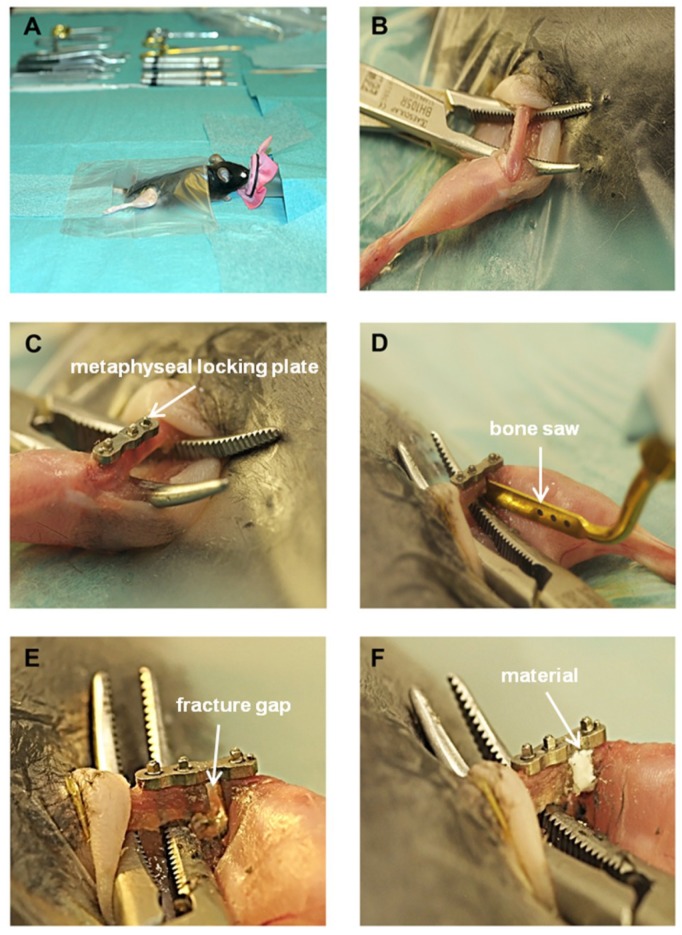
Phases of the surgical approach. (**A**) anesthetized mouse on the operating table, (**B**) exposure of the femur, (**C**) attached metaphyseal locking plate, (**D**) process of osteotomy, (**E**) 1.2 mm fracture gap after osteotomy, (**F**) implanted paste-like cement/MBG composite. White arrows in images C, D, E and F point at the metaphyseal locking plate, bone saw, fracture gap and material, respectively.

Prior to surgery mice were injected subcutaneously with 0.1 mg/kg bodyweight buprenorphine (Buprenodale^®^, Dechra, Staffordshire, UK) and anesthetized by isoflurane CP^®^ (CP-Pharma, Burgdorf, Germany) inhalation via an anesthesia machine (Groppler, Deggendorf, Germany). Fur was covered with sterile drape (Figure 8A) before skin and *Fascia lata* were incised. The femur was exposed by carefully thrusting aside musculature and placing a hemostat underneath the femur (Figure 8B). The metaphyseal MouseFix system (RISystem AG, Davos, Switzerland) was used for stabilizing the metaphyseal part of the right femur prior to osteotomy. Metaphyseal locking plate was placed with the concave side onto the anterolateral region of the right femur as described by Histing et al. (2012) [44], shown in Figure 8C. A bicortical osteotomy of 1.2 mm was conducted using a Piezosurgery^®^ osteotomy bone saw equipped with an OT7S-3 saw blade (Mectron, Cologne, Germany), (Figure 8D,E). The fracture gap was then completely filled with the bone cement/MBG composite with or without BDNF (Figure 8F). Thereafter, musculature and skin were stitched with Vicryl 6-0 suture material (Ethicon, Somerville, NJ, USA). Mice were sacrificed 35 days after surgery and the right femur, tibia and blood collected to be analyzed. In total 87 mice underwent surgery.

### 4.4. Real-Time Reverse Transcriptase Polymerase Chain Reaction (Real-Time RT-PCR)

RNA was isolated by homogenizing tissue from the implant interface of the right femur using the Mixer Mill MM400 (Retsch, Haan, Germany) and the miRNeasy Mini Kit (Qiagen, Hilden, Germany) according to the manufacturer’s protocol. For mRNA anaylsis*,* 750 ng of RNA were reverse transcribed into cDNA using the Quantitect Reverse Transcription Kit (Qiagen, Hilden, Germany) and a cycler (TC-3000, Techne, Bibby Scientific, Burlington, NJ, USA). The mRNA expression of alkaline phosphatase (Alp), cathepsin K (Ctsk) and connexin 43 (Cx43) was analyzed by real-time RT-PCR using the Quantifast SYBR Green PCR Kit (Qiagen, Hilden, Germany) and a LightCycler 2.0 (Roche, Basel, Switzerland). Primers used are listed in Table 1. Purity of the RT-PCR products was verified by a melting curve. Negative controls were not reverse transcribed or received water instead of cDNA. Results were evaluated using delta threshold cycle (CT) values with reference to the housekeeping gene beta-actin.

For microRNA analysis 1000 ng of RNA were reverse transcribed into cDNA using the miScript II RT Kit (Qiagen, Hilden, Germany) according to the manufacturer’s protocol. For real-time RT-PCR analysis the Quantitect SYBR Green PCR-Mastermix (Qiagen, Hilden, Germany) and miScript Primer assays (Qiagen, Hilden, Germany) as well as the Lightcycler 2.0 (Roche, Basel, Switzerland) were used. MicroRNAs analyzed were mmu-miR-335-5p (Accession No. MIMAT0000766), mmu-miR-132-3p (Accession No. MIMAT0000144), mmu-miR-148a-3p (Accession No. MIMAT0000516), mmu-miR-503-5p (Accession No. MIMAT0003188) and mmu-miR-376b-5p (Accession No. MIMAT0003388). For evaluation, delta cycle threshold (CT) values were analyzed using the reference gene RNU6-6p (pseudogene, 107 bp, Accession No. NR_002752.2).

### 4.5. Micro-CT

Scans were carried out with the micro-CT System Skyscan 1173 (Bruker microCT, Kontich, Belgium). Femurs were scanned with a tube voltage of 100 kVp and a tube current of 80 µA. The image pixel size was 7.11 µm (nominal isotropic resolution). All samples were scanned over 360° in rotation steps of 0.2° and a 6-fold frame averaging for noise reduction. To reduce image artifacts, a beam hardening was carried out with an aluminum filter of 1 mm thickness. Cross sectional images of the same femur were reconstructed using the NRecon (Version 1.7.0.4, Bruker microCT) based on a cone-beam reconstruction algorithm as described by Feldkamp et al. (1984) [45]. A Gaussian kernel (*σ* = 2) was taken for image noise reduction. The 3D volume rendering was performed using the ANALYZE Software package (ANALYZE 12, Mayo Clinic, Rochester, MN, USA).

### 4.6. Histological Analyses of Sections Embedded in Technovit 9100

Femur sections of 5 µm, embedded in Technovit 9100 [46] were deplastified and rehydrated prior to histological analyses. To distinguish between granulation tissue, cartilage and newly formed mineralized bone Movat pentachrome staining was performed as described by Olah et al. (2000) [47]. Osteoclasts were visualized by TRAP histochemistry as described in detail previously [46]. Osteoblasts were visualized by toluidine blue stain [46]. To demonstrate osteoid formation and mineralization von Kossa/van Gieson staining was performed as described earlier [46]. For visualization of blood vessels ASMA immunohistochemistry was applied. Endogenous peroxidase was blocked using 3% H_2_O_2_ before incubation with the primary ASMA antibody (Acris, Herford, Germany; dilution: 1:100) over night at 4 °C. Secondary goat-anti-rabbit antibody (Vector, Burlingame, CA, USA) was added for 30 min (dilution: 1:500). Then, Vectastain Elite ABC horseradish peroxidase (Vector, Burlingame, CA, USA) was added for 30 min, followed by incubation with NovaRed (Vector, Burlingame, CA, USA) substrate for 5 min and counter staining with hematoxylin (Thermo Fisher Scientific, Waltham, MA, USA) for 20 s. Finally, sections were dehydrated and cover slipped using DePex (Serva, Heidelberg, Germany).

### 4.7. Histomorphometrical Analysis of Fracture Gap and Callus Tissue

Images of sections were captured using a Leica DM5500 microscope (Leica, Wetzlar, Germany) equipped with a DFC7000 T camera and the Leica Application Suite X (LASX) computer software version 3.3.3.16958. A Zeiss microscope (Zeiss, Oberkochen, Germany) equipped with a Leica DC500 camera (Leica, Wetzlar, Germany) and the computer programs IM 1000 (version 4.0, Leica Microsystems Imaging Solutions, Ltd., Cambridge, UK) as well as Adobe Photoshop CS3 (Adobe Systems, Munich, Germany) were used to quantify the area of granulation tissue, cartilage and newly formed bone in relation to total region of interest (ROI). ROI comprised of the implant-filled fracture gap between the distal and middle screws and callus tissue which was marked manually using Adobe Photoshop and calculated in mm^2^. The ratio of granulation tissue, cartilage and newly formed bone to the whole ROI was calculated in percent. Analyses were conducted on one section per animal (M3 mAChR KO: *n* = 5 and WT: *n* = 5).

### 4.8. ToF-SIMS, HR-SEM and EDS

The surfaces of bone sections were analyzed by ToF-SIMS and HR-SEM. All ToF-SIMS measurements were carried out with a TOF.SIMS 5-100 machine (IONTOF GmbH, Muenster, Germany). For analysis 25 keV Bi_3_^+^ primary cluster ions were used and the primary ion gun was operated in high-current bunched mode to obtain the best available mass resolution. The full width at half resolution (fwhm) was *m*/Δ*m* > 4000 for Ca^+^ for the complete scan. Charge compensation was done with low energetic electrons. For imaging stage scans were carried out with a patch size of 400 × 400 µm^2^. Each patch was scanned 10 times with 2 shots per pixel and a pixel density of 120 pixel/mm. In total 12 scans with a primary ion current of 0.28–0.42 pA were recorded. For data evaluation we used the SurfaceLab 6.7 (IONTOF GmbH, Muenster, Germany) software. Detail images were taken in low current bunched mode with a lateral resolution of approximately 2 µm.

Scanning electron microscopy (SEM) analysis was carried out with a Zeiss Merlin microscope (Zeiss, Oberkochen, Germany) and EDS analysis with an x-max 50 mm^2^ detector (Oxford Instruments, Abingdon, UK) at 5 mm working distance and 10 kV acceleration voltage. In advance a thin carbon layer was deposited with an Edwards Scancoat Six to avoid sample charging. Data evaluation was done with the Aztec software from Oxford instruments.

### 4.9. FACS

Leukocyte analysis in blood was performed as previously described [48]. For determination of distinct leukocytes the following antibodies were used: Fluorescein isothiocyanate (FITC)-anti-mouse/human CD11b, Phycoerythrin (PE) anti-mouse CD3ε, PE/Cy7 anti-mouse CD4, Peridinin-Chlorophyll-protein (PerCP)/Cy5.5 anti-mouse CD19, Allophycocyanin (APC) anti-mouse CD8a, APC/Cy7 anti-mouse CD45, Pacific Blue anti-mouse NK-1.1, Brilliant Violet 510 anti-mouse Ly 6G/Ly 6C (Gr-1) (all from BioLegend, San Diego, CA, USA).

### 4.10. CRP Analysis

Heparinzed blood was centrifuged for 10 min at 3500 rpm and 4 °C to obtain plasma. CRP concentrations in plasma were determined using the ProcartaPlex^TM^ Multiplex Immunoassay (Affymetrix eBioscience, Thermo Fisher Scientific, Waltham, MA, USA) according to the manufacturer’s protocol. In brief, beads were vortexed and added to a ProcartaPlex 96-well flat bottom plate. The plate was inserted into a Hand-Held Magnetic Plate Washer to allow beads to accumulate on the bottom before supernatant was removed. Beads were then washed and plasma samples added to each well. The plate was sealed, removed from the plate washer and covered with a black microplate lid. Subsequently, the plate was shaken for 120 min at 500 rpm. Samples were washed as described above before detection antibody mixture was added and shaken for 30 min at 500 rpm. After washing, Streptavidin-PE was added for 30 min at 500 rpm, samples washed again and reading buffer applied for 5 min at 500 rpm. Analysis was performed using a Becton Dickinson (BD) FACS Canto II (Becton Dickinson Biosciences, Franklin Lakes, NJ, USA).

### 4.11. ALP Analysis in Callus Tissue Cultured In Vitro

Callus tissue was cut into 1 mm pieces and rinsed in 5 mL Hibernate A medium (Gibco, Waltham, MA, USA) + 10% fetal calf serum (FCS; PanSera ES, Pan Biotech, Aidenbach, Germany) + 0.2% Gentamicin/Amphothericin (Gibco, Waltham, MA, USA). It was degraded in 5 mL of warmed digestion buffer consisting of Hibernate A medium (Gibco) equipped with 400 U/mL collagenase (Wako chemicals, Neuss, Germany) and 0.3 mM CaCl_2_ for 2 h at 37 °C. Every 30 min digestion buffer was renewed. Digestion process was stopped by applying Hibernate A medium + 10% FCS and samples filtered using a 70 µm cell strainer (Becton Dickinson Falcon, Franklin Lakes, NJ, USA). Samples were centrifuged for 5 min at 500× *g* and 4 °C, rinsed twice in Hibernate A medium (Gibco, Waltham, MA, USA) + 10% FCS + 0.2% Gentamicin/Amphothericin (Gibco, Waltham, MA, USA) and cell number determined. Finally, 10,000 cells/cm^2^ were seeded in Petri dishes, incubated at 37 °C and medium exchanged after 5 days.

For the analysis of differentiation of murine MSCs into osteoblasts, concentration and activity of ALP were measured after 0, 3 and 6 days of in vitro culture using the SensoLyte pNPP Alkaline Phosphatase Assay Kit (AnaSpec, Fremont, CA, USA) according to the manufacturer’s protocol. In brief, after washing with PBS cells were lyzed in 1% triton-X-100 (Sigma, St. Louis, MO, USA) and immediately stored at −80 °C. Prior to analysis samples were thawed on ice, centrifuged for 4 min at 250× *g* and 4 °C and buffer pipetted in a 96-well-plate (Greiner bio-one, Kremsmuenster, Austria) before samples, standard and blank were added. Subsequently, para-nitrophenylphosphate substrate was added. ALP activity was measured at 405 nm and 37 °C using the Synergy HT plate reader (BioTek, Winooski, VT, USA). ALP concentration and activity were referenced to total protein obtained from a Bio-Rad DC^TM^ Protein Assay (Bio-Rad Laboratories, Inc., Hercules, CA, USA) that was performed according to the manufacturer’s protocol in the same cell lysates mentioned above. Absorbance was measured at 750 nm using a Synergy HT plate reader (BioTek, Winooski, VT, USA).

### 4.12. Statistical Analysis

Statistical analysis and generation of graphs were performed using the statistics program SPSS (version 22.0; SPSS Institute Inc., Chicago, IL, USA). Real-time RT-PCR delta CT values, Micro-CT and histomorphometrical results, CRP concentrations, FACS- and ALP assay results were analyzed with Kolmogorov-Smirnow-, T-, Mann-Whitney- and Kruskal-Wallis-tests. Sample size (*n*) ranged from 5–8 mice. A value of *p* ≤ 0.05 was regarded as significant.

## 5. Conclusions

We were able to establish the first metaphyseal fracture healing model for testing pasty bone substitute materials in osteoporotic bone of mice. We therefore consider future studies on metaphyseal fracture healing in mice with application of bone substitute materials as feasible and especially valuable in respect to osteoporosis research.

BDNF might either have different effects in osteoporotic and non osteoporotic bone or higher concentrations are necessary for enhanced fracture healing in osteoporosis which should be addressed in future studies. However, in non osteoporotic bone the pasty BDNF-functionalized cement/MBG composite was beneficial for fracture healing. Moreover, the BDNF-functionalized cement/MBG composite decreased leukocyte numbers within a physiological range in M3 mAChR KO mice suggesting anti-inflammatory effects and consequently biocompatibility during fracture healing in osteoporosis.

## Figures and Tables

**Table 1:** Mouse primers used for mRNA analysis by real-time RT-PCR.

Primer	Sequence	Length (bp)	Accession No.
Alp for rev	TCAGCTAATGCACAATATCAAGG TCCACATCAGTTCTGTTCTTCG	87	NM_007431.2
Ctsk for rev	GAGGCGGCTATATGACCACT CTTTGCCGTGGCGTTATACA	119	NM_007802.3
Cx43 for rev	TGCTTCCTCTCACGTCCCAC CGCGATCCTTAACGCCCTTG	127	NM_010288.3
ß-actin for rev	TGTTACCAACTGGGACGACA GGGGTGTTGAAGGTCTCAAA	165	NM_007393.3

## Abbreviations

α-TCP	α-tricalcium phosphate
ALP	Alkaline phosphatase
ASMA	Alpha smooth muscle actin
BDNF	Brain-Derived Neurotrophic Factor
BMP	Bone morphogenic protein
BV	Bone Volume
BV/TV	Bone volume fraction
CPCs	Calcium phosphate cements
CRP	C-reactive protein
EDS	Energy-dispersed X-ray spectroscopy
HA	Hydroxyapatite
HR-SEM	High-resolution scanning electron microscopy
KO	Knockout
M3 mAChR	M3 muscarinic acetylcholine receptor
MBG	Mesoporous bioactive glass
Micro-CT	Micro-computed tomography
MSCs	Mesenchymal stem cells
RT-PCR	Reverse-transcription polymerase chain reaction
TGF-β	Transforming growth factor
ToF-SIMS	Time-of-Flight Secondary Ion Mass Spectrometry
TRAP	Tartrate-resistant acidic phosphatase
TV	Total Volume
VEGF	Vascular endothelial growth factor
WT	Wild type
